# Stable carbon isotope analyses of nanogram quantities of particulate organic carbon (pollen) with laser ablation nano combustion gas chromatography/isotope ratio mass spectrometry

**DOI:** 10.1002/rcm.7769

**Published:** 2016-11-28

**Authors:** Linda van Roij, Appy Sluijs, Jelmer J. Laks, Gert‐Jan Reichart

**Affiliations:** ^1^ Department of Earth Sciences, Faculty of GeosciencesUtrecht UniversityHeidelberglaan 2, 3584 CS UtrechtThe Netherlands; ^2^ Royal Netherlands Institute for Sea Research (NIOZ)Landsdiep 4, 1797 SZ ‘t Horntje (Texel)The Netherlands

## Abstract

**Rationale:**

Analyses of stable carbon isotope ratios (*δ* ^13^C values) of organic and inorganic matter remains have been instrumental for much of our understanding of present and past environmental and biological processes. Until recently, the analytical window of such analyses has been limited to samples containing at least several μg of carbon.

**Methods:**

Here we present a setup combining laser ablation, nano combustion gas chromatography and isotope ratio mass spectrometry (LA/nC/GC/IRMS). A deep UV (193 nm) laser is used for optimal fragmentation of organic matter with minimum fractionation effects and an exceptionally small ablation chamber and combustion oven are used to reduce the minimum sample mass requirement compared with previous studies.

**Results:**

Analyses of the international IAEA CH‐7 polyethylene standard show optimal accuracy, and precision better than 0.5‰, when measuring at least 42 ng C. Application to untreated modern Eucalyptus globulus (C_3_ plant) and Zea mays (C_4_ plant) pollen grains shows a ~ 16‰ offset between these species. Within each single Z. mays pollen grain, replicate analyses show almost identical *δ* ^13^C values.

**Conclusions:**

Isotopic offsets between individual pollen grains exceed analytical uncertainties, therefore probably reflecting interspecimen variability of ~0.5–0.9‰. These promising results set the stage for investigating both *δ* ^13^C values and natural carbon isotopic variability between single specimens of a single population of all kinds of organic particles yielding tens of nanograms of carbon. © 2016 The Authors. *Rapid Communications in Mass Spectrometry* Published by John Wiley & Sons Ltd.

Stable carbon isotopic ratios (*δ* ^13^C values) measured on organic material are used to determine the isotopic signature of carbon sources, investigate carbon partitioning between reservoirs and investigate processes resulting in fractionation during biosynthesis. Today, the *δ* ^13^C signatures of blood cells, plant and animal tissue, bacterial cells, algal cells, pollen, fungal spores, crustaceans, chironomids and many other organic particles are pivotal in many geological and biological studies.^1^, ^2^, ^3^, ^4^, ^5^, ^6^, ^7^, ^8^ For plants, it has been shown that these signatures reveal isotopic variability between species, specimens and organs resulting from differences in climatic conditions, harvest time, material composition, photosynthetic pathways and secondary metabolism.^9^, ^10^, ^11^, ^12^, ^13^, ^14^, ^15^, ^16^, ^17^, ^18^

High precision and accurate measurements of the *δ* ^13^C values of micrometer‐sized organic particles are currently limited to secondary ion beam techniques.^19^ These techniques are, due to substantial processing time and relatively rough surfaces on the nanometer scale, however, not (yet) suitable for standardized analyses of large numbers of entire single particles. An alternative approach is based on measuring the *δ* ^13^C values of extracted organic compounds and critically relies on the availability of specific molecules related to specific organisms.^20^ Most compounds, however, are related to groups of organisms rather than to specific species and therefore inevitably integrate the isotopic signature of whole communities.^21^, ^22^, ^23^

Measurements of solid organic matter *δ* ^13^C values are typically based on the combustion of organic matter through an elemental analyzer (EA), after which the produced CO_2_ is transported by a carrier gas and measured by isotope ratio mass spectrometry (IRMS). Samples are combusted in sample cups in the presence of oxygen and require typically ~25 μg of carbon.^24^ The minimum amount of carbon needed is related to the blank associated with the cup and the sample carousel. Over the last decade, this blank has been reduced by cleaning protocols and low‐blank autosamplers.^25^, ^26^, ^27^, ^28^, ^29^ To decrease the mass of carbon required for a typical EA/IRMS measurement, Polissar *et al*.^28^ further adjusted a conventional EA by decreasing the EA to isotope ratio mass spectrometer split ratio and by using a cryotrap. This allowed them to measure *δ* ^13^C values on ~0.5 μg of carbon of international and in‐house standards of various origins (sucrose, oil, sediments) with 1‰ precision (2*σ*). A so‐called spooling wire microcombustion (SWiM) device described by Sessions *et al*.,^30^ based on earlier prototypes,^31^, ^32^ and improved by Eek *et al*.,^33^ was designed to measure the *δ* ^13^C values of soluble nonvolatile organic compounds.^34^, ^35^, ^36^ A precision better than 1‰ for samples containing at least 10 ng of carbon (2*σ*), and an accuracy of 0.5‰, can be obtained. Despite the device being designed for the study of soluble organic molecules, pollen grains have also been analyzed by SWiM/IRMS.^37^ In such studies, the precision and accuracy are sufficient to distinguish between sediment samples containing pollen with the isotopic signature of only C_3_ plants and samples containing also C_4_ plants. The *δ* ^13^C values of C_3_ plants typically range between −34‰ and −24‰, whereas those of C_4_ plants range between −19‰ and −6‰.^18^ Higher accuracy and precision than indicated in the study by Nelson *et al*.^37^ are required when studying more subtle differences in biologically and environmentally influenced *δ* ^13^C signatures, as expected to be found in different plant functional groups such as herbaceous, deciduous woody, and evergreen woody plants,^10^ and in various types of unicellular organisms in terrestrial and marine ecosystems, and when investigating isotopic variability within assemblages of organic particles such as pollen and dinoflagellate cysts.

Laser ablation (LA) techniques in combination with continuous‐flow IRMS can provide an alternative approach to measuring *δ* ^13^C values on small solid samples. Without the use of carrier material such as cups or wires, samples can also be smaller than for EA/IRMS and SWiM due to decreased introduction of contaminating carbon. The first setups for LA/IRMS used a CO_2_ laser and a cryotrap to analyze carbonates and phosphates.^38^, ^39^, ^40^ The introduction of a combustion oven^41^ was followed by the use of Nd:YAG lasers^42^, ^43^ allowing for the analysis of organic matter. Analysis of the international cellulose IAEA CH‐3 standard and bamboo stem segments using such a Nd:YAG laser ablation system coupled to a combustion oven and a GC/IRMS instrument yielded a 0.2–0.3‰ accuracy and a 0.2‰ precision.^43^ However, at least 20 μg of sample (~9 μg C) was required.

More recently, Moran *et al*.^42^ managed to analyze samples down to 65 ng C with both an accuracy and a precision of 0.1–0.3‰ (1*σ*). Their study and other previous attempts to measure small aliquots of carbon using lasers were based on more traditional 266 nm laser systems. A 193 nm DUV (deep ultraviolet: family of lithographic wavelengths)^44^ ArF excimer laser is, however, superior in generating (small) particles, especially on organic matter,^45^, ^46^ with less isotopic fractionation between particles during ablation.^47^

Here we present a newly developed method combining laser ablation, nano combustion gas chromatography and isotope ratio mass spectrometry (LA/nC/GC/IRMS) using a 193 nm DUV ArF excimer laser. Furthermore, a dedicated ablation chamber was designed, reduced in size compared with the one used by Moran *et al*.,^42^ to prevent dilution following ablation. We test the analytical performance of the system with a polyethylene standard and apply it by measuring the *δ* ^13^C values of single modern pollen grains of a C_3_ and a C_4_ plant. Data are compared with those from conventional EA/IRMS analyses of the standard and multispecimen pollen grains.

## Experimental

### System setup

The nano combustion device is based on a continuous gas flow system, consisting of an ablation chamber positioned under a 193 nm DUV ArF laser system (COMPex 102; Lambda Physik, Göttingen, Germany), coupled online via a GC combustion III interface (ThermoFinnigan, Bremen, Germany) to a Delta V Advantage isotope ratio mass spectrometer (ThermoFinnigan) (Supplementary Fig. S1, Supporting Information). The custom‐built miniaturized ablation chamber consists of an aluminum cylinder (50 mm diameter × 23 mm length) with a hollow space for the samples (3 mm diameter × 6.5 mm length, 45 μL volume) (Supplementary Figs. S1 and S2, Supporting Information). A nickel disc (6 mm diameter) is locked against the sample space from below, serving as a relatively ablation‐resistant surface.^48^ The top of the sample cavity is closed by a sapphire glass plate (50 mm diameter × 0.4 mm thickness), which is attached to two O‐rings by means of a small suction pump creating a vacuum between the rings. This vacuum between the ablation chamber and outside prevents leakage of atmospheric gasses into the chamber via the O‐rings. The gas inflow and outflow of the chamber are positioned at the sides, using stainless steel tubing (1 mm OD, 0.7 mm ID), glued into the chamber. The actual gas flow is through two fused‐silica capillaries of 0.32 mm i.d., each ~2 m in length, mounted to the steel tubes using 15% graphite/85% Vespel™ ferrules.

The pressure is set at 0.5 bar above atmosphere throughout the system at all times. Standards and samples are ablated using deep UV laser light at various energy levels. The resulting particles are carried on a (BIP5.0) helium flow through the fused‐silica capillaries and oxidized at 940°C in a combustion oven, filled with oxidized copper and nickel wires. The oven is oxidized monthly by admixing O_2_ to the helium flow, with the gas flow bypassing the IRMS instrument. The produced CO_2_ flows into a PoraBOND Q column (25 m, 0.32 mm, 5 μm P/N:CP7351; Agilent Technologies, Santa Clara, CA, USA), through a GC combustion III interface (ThermoFinnigan) with open split (reduction oven is bypassed) and into the IRMS instrument. Approximately 0.9 mL/min of helium containing the sample flows into the open split, whereas a constant flow of 0.3 mL/min continues into the IRMS instrument. As a result of this open split ratio, ~33% of the sample enters the instrument and thus represents the maximum possible amount being available for ionization for isotope ratio analysis.

### Blank and sample analysis

The LA/nC/GC/IRMS system does not suffer from blank values introduced by sample containers or cryotrapping. However, the gases used for continuous flow do contain minute amounts of CO_2_ and/or CO_2_ may leak into the gas flow through small imperfections in the gas tight fittings. To determine the potential effect of such contaminants, CO_2_ was trapped three times for 3000 s and analyzed for its *δ* ^13^C value. Since ablation of the nickel base of the ablation chamber could potentially also contaminate signals in the highly sensitive setup used here, we ablated an empty sample tray at 1.2 J/cm^2^.

**Figure 1:**
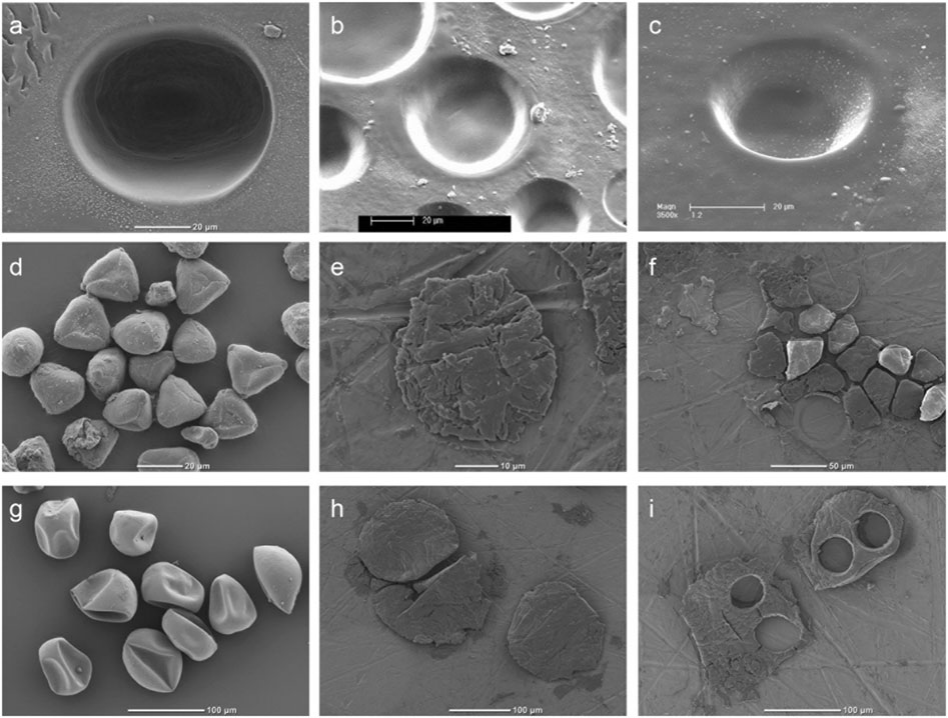
SEM images of the samples. PE foil: crater (a), several craters of various diameters and depths in PE foil tilted at a 40° angle (b–c). Eucalyptus globulus pollen: untreated grains (d), pressed grain (e), several pressed grains and three ablation craters (f). Zea mays pollen: untreated grains (g), pressed grains (h), two pressed grains with each two ablation craters (i).

To test the viability of the newly developed LA/nC/GC/IRMS method, we measured the international IAEA CH‐7 polyethylene standard (PE), which has a certified *δ* ^13^C value of −32.151 ± 0.050‰ (1*σ*), as expressed relative to the international Vienna Pee Dee Belemnite carbonate standard (VPDB).^49^ Using PE as an internal standard we measured the *δ* ^13^C values of untreated modern pollen grains of both Eucalyptus globulus (Greer Labs Inc., Lenoir, NC, USA) and Zea mays (field sample, southern USA; collection of Laboratory of Palaeobotany and Palynology, Utrecht University, The Netherlands) (Figs. 1(d) and 1(g)).

For comparison, we carried out traditional *δ* ^13^C signature measurements on E. globulus pollen (30–50 μg) and Z. mays pollen (34–55 μg) using an elemental analyzer (NA1500 NCS; Fisons, Loughborough, UK) coupled online to a Delta plus IRMS instrument (ThermoFinnigan), including PE (20–32 μg) as a reference. These EA/IRMS analyses, from here on referred to as ‘bulk’, were calibrated using USGS‐24 graphite (−16.05‰), an in‐house sediment standard ‘GQ’ (−26.68‰) and NBS‐22 oil (−30.03‰).

We tested the sensitivity of the LA/nC/GC/IRMS system using variable laser settings. Pieces of PE foil were placed on a nickel disc in the ablation chamber and ablated using a range of crater sizes (20–120 μm), a range of durations (1–10 s) and a range of energy levels (0.9–2.7 J/cm^2^), all at a repetition rate set at 20 Hz (Fig. 1(a)). The resulting ^13^C/^12^C ratios were initially compared with that of a CO_2_ reference gas, which prior to LA/nC/GC/IRMS analyses was calibrated off‐line to an internal house standard (Naxos, which is turn was calibrated to NBS‐18 and NBS‐19 international standards), with a *δ* ^13^C value of −36.1‰. A drift correction was performed when the R^2^ for the linear regression between the *δ* ^13^C values of the reference gas and the corresponding retention time of the peak was better than 0.6. The peak sizes are expressed in volt seconds (Vs) for the cumulative signals of *m/z* 44, 45 and 46. The *δ* ^13^C values of PE were not corrected for their certified value in order to study potential drift in values due to unstable reference gas *δ* ^13^C values and to prevent averaging out of the standard deviation.

For 131 ablations, the crater size and its position on the PE foil were tracked so that the corresponding depth of the ablated crater could be determined by tilting the foil by 40° using scanning electron microscopy (SEM) (XL30 SFEG; FEI/Philips, Hillsboro, OR, USA; Figs. 1(b) and 1(c)). A dedicated imaging software package (measureIT, supplied by Olympus Soft Imaging Solutions, Münster, Germany^50^) was used to determine crater depths, which were used to calculate the amount of ablated carbon and subsequently compared with the corresponding peak area detected by the IRMS instrument.

Pollen grains of E. globulus and Z. mays were placed between two nickel discs in a hydraulic press, and pressed together at 3.5 ton/cm^2^ (357 MPa), to adhere specimens to the Ni surface (Figs. 1(e) and 1(h), respectively). Separated discs with samples adhered to them were placed sequentially in the ablation chamber, after which the chamber was flushed for ~30 min until the Ar and CO_2_ signals returned to stable background values, before ablating. The laser settings were adjusted to minimum energy (~0.9 J/cm^2^) when ablating the pollen grains, to avoid loss of specimens during the first few laser pulses. E. globulus was analyzed as single pollen grains (Fig. 1(f)), and as multiple (2–5) pollen grains. Z. mays, which is much larger, was measured once or two or three times per single pollen grain (Fig. 1(i)).

Reference to the VPDB scale was achieved through bracketing by the international IAEA CH‐7 PE foil as the only standard. The ablation duration, crater size and energy level were adjusted (usually 3 s, 80 μm and 25 kV) during PE measurements so that at least 4 Vs peaks would be obtained for optimal precision (see discussion below). Every run started with three reference gas analyses, followed by 2× PE standards, 2× pollen samples, 1× PE, 2× pollen samples, 1× PE, 2× pollen samples, and 1× PE, and ending with three reference gas analyses. As a result, 6 pollen samples and 5 PE standards could be analyzed in a 1‐h run. During some runs, however, samples were skipped due to loss of pollen early during ablation. Runs succeed one another instantly as long as there is still pollen left on the same nickel disc, which can easily hold enough for an entire day's worth of measurements. After each run, the *δ* ^13^C sample values were calibrated to the PE standard to correct for potential drift and offsets. A drift correction was performed when the R^2^ for the linear regression between the *δ* ^13^C values of the PE standard and the retention time of the peaks was better than 0.6. Subsequently, the offset was corrected for by using the difference between the average of all PE data within a 1‐h run and the certified *δ* ^13^C value of PE. The values are given in mean ± the standard error of the mean (SE), unless stated otherwise. The standard deviation is indicated as 1*σ* or 2*σ*, depending on the discussed data.

## Results and Discussion

### Background and blanks

The stable carbon isotopic value of the CO_2_ contaminating the measurements, even when only minor, is essential in evaluating potential analytical offsets. This residual CO_2_ in our system revealed a *δ* ^13^C value of −26.87 ± 0.08‰ (1*σ* = 0.13‰), which is close to previous observations by Moran *et al*.^42^ (−28.64‰, 1*σ* = 2.61‰) and remarkably also to those of Polissar *et al*.^28^ (−27.76 ± 1.26‰), whose EA/IRMS setup is very different from the LA/IRMS systems. The seemingly similar carbon source in all different systems does, however, not correspond with atmospheric CO_2_ (at about −8.2‰)^51^ leaking into the system. Since trapping CO_2_ for 3000 s results in a cumulative peak area of only 2.41 ± 0.04 Vs, a standard analysis without trapping and generating peak widths (far) below 100 s should not be biased by more than 0.08 Vs.

The laser beam does not reach the nickel during ablation of PE, since the foil is relatively large and thick. Formation of the ablation crater at the surface of the foil can clearly be observed from the magnified view on the monitor of the laser ablation system. However, ablation of the nickel sample disc could potentially influence pollen analyses. Ablation of an empty nickel sample tray shows only tiny peaks of 0.07 Vs, with an inconsistent *δ* ^13^C value of −30.74 ± 1.00‰ (1*σ* = 6.43‰, *n* = 41). The carbon detected might have been incorporated in the nickel during fabrication, with the large internal variability hinting at an inhomogeneous distribution and/or fractionation during the release of carbon from the nickel. However, the absolute amounts of carbon released are very small.

### Testing LA/nC/GC/IRMS performance using a PE standard

The CO_2_ yield, after conversion of the ablated material in the combustion oven, is generally similar for PE and pollen. A typical peak shape for PE and pollen is demonstrated in Supplementary Fig. S3 (Supporting Information). In this particular analysis the total peak area varies between 5.5 and 6.2 Vs for PE and is 2.4 and 5.2 Vs for Z. mays. A clear correlation is observed between peak intensity (height) and duration (width), so that (much) smaller and larger CO_2_ signals show a similar shape. This suggests that there is no appreciable loss from fronting or bypassing of material in the combustion oven. No further concentration of the signal by adding a cryotrap is necessary due to the use of the small custom‐built sample chamber. Since cryotrapping generally increases background signals as well, we thereby reduced the analytical blank.

Considering the limited duration of the ablation itself (1–10 s), appreciable tailing is observed and hence care has to be taken to avoid overlap between analyses. Tailing may result from particles that are not transported instantaneously or exhibit delayed reaction in the combustion oven. Theoretically, the chamber volume of 45 μL is flushed (helium at a flow of 0.9 mL/min) in 3 s. Assuming perfect mixing inside the ablation chamber, it will take hence 14 and 28 s to flush 99 and 99.99% of the initial gas, respectively. However, particles probably will linger in less‐well flushed parts of the chamber, adding to the apparent particle residence time. Besides the formation of particles, ablation may lead to direct volatilization of gaseous CO_2_, even with a 193 nm laser. Moran *et al*.,^42^ however, found that gaseous CO_2_ resulting from the ablation of a fishing line with a 266 nm CO_2_ laser did not exceed trace levels. Since using a 193 nm excimer laser should further reduce the production of volatiles,^46^ we do not consider this to be an issue for the current setup. Most likely, tailing results from the time involved with the reactions in the combustion oven^52^ or subsequent column overload,^24^ although the latter is not evident from the peak shape observed. Monitoring yield and peak shape, the open split ratio was subsequently optimized at 33% of the gas being sampled going to the IRMS instrument.

**Figure 2:**
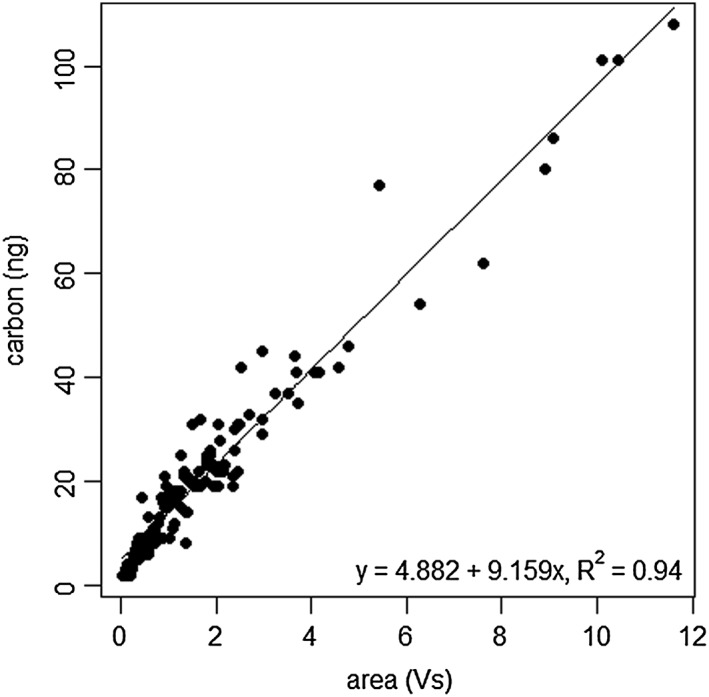
Amount of carbon in PE versus generated cumulative peak area of *m/z* 44, 45 and 46. Mass in ng C, peak area in Vs.

The international IAEA CH‐7 PE standard is homogeneous on the submilligram scale,^49^ but has to our knowledge never been analyzed on the nanogram scale. The PE standard was analyzed 534 times using a variable crater size, ablation duration and energy level. For 131 of those PE measurements the mass of ablated carbon (C) was calculated using known crater diameters, measured crater depth, a PE density of 0.92 g/cm^3^ and a carbon content of 80%, and subsequently compared with the corresponding peak area in order to determine linearity of the system and relative integrated yield (Fig. 2). The observed strong linear correlation shows that for smaller and larger samples equal fractions are turned into CO_2_ and thus that the capacity of the combustion oven was never limiting. The correlation shows an integrated system sensitivity of 9.1 ng C/Vs. This is similar to the 6.4 ng C/Vs reported by Moran *et al*.^42^ and 9 ng C/Vs (peak of *m/z* 44 only) by Nelson.^53^ Moran *et al*.,^42^ however, used cryotrapping, which also increases blanks.

The total of all 534 PE standard analyses performed by LA/nC/GC/IRMS show *δ* ^13^C values of −32.24 ± 0.04‰ (1*σ* = 0.98‰), which is 0.09‰ more depleted in ^13^C compared with the accepted *δ* ^13^C value (Supplementary Table S1, Supporting Information). Cross checking by analyzing larger pieces of the PE standard using EA/IRMS results in slightly higher values with −32.08 ± 0.06‰ (1*σ* = 0.12‰, *n* = 5), in line with the certified value.

**Figure 3:**
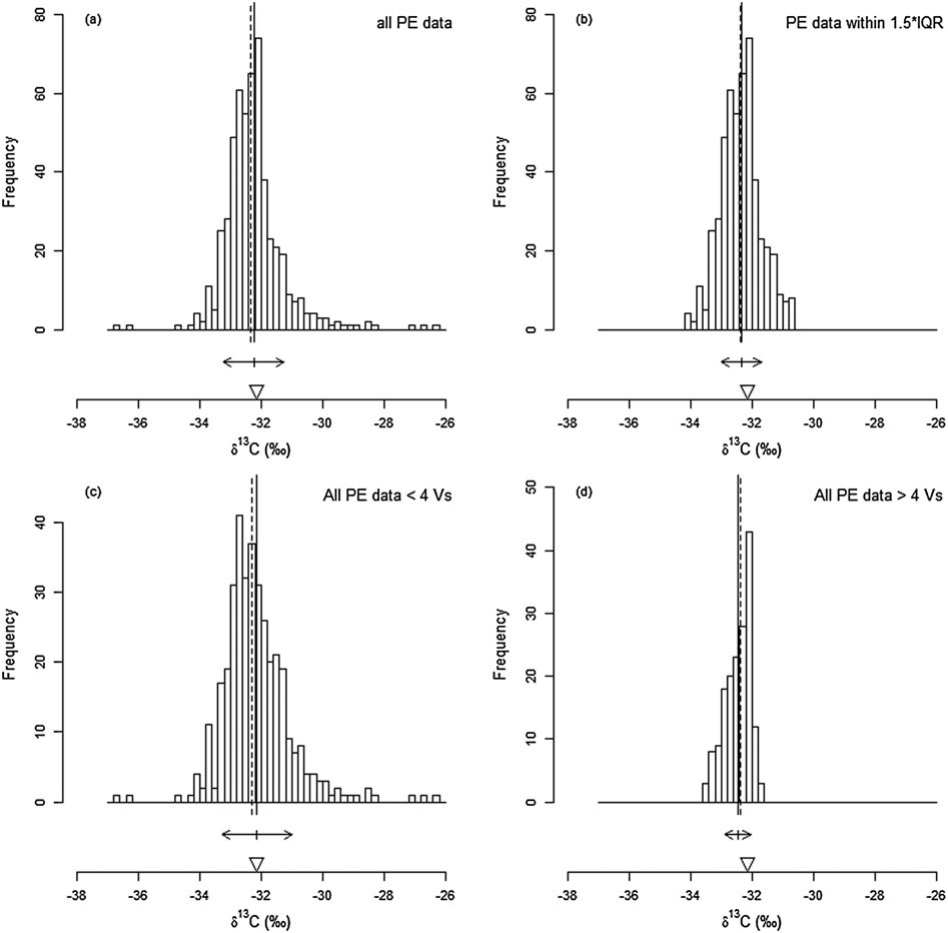
Histograms of *δ* ^13^ C values of PE data resulting from LA/nC/GC/IRMS analyses and data subsets. All PE data (a) and PE data within 1.5 times the interquartile range (b), PE data within 2 *σ* ; not shown. All PE data <4 Vs (c) and all PE data >4 Vs (d). Vertical line indicates the mean, dashed line indicates the median. Arrows indicate the standard deviation of the mean. Triangle indicates certified value of PE.

The distribution of isotopic values measured using LA/nC/GC/IRMS shows appreciable tailing towards both heavy and light values (Fig. 3(a)). Common approaches regarding outliers are to eliminate data falling either outside 2*σ* or outside 1.5 times the interquartile range (IQR). The first approach (Supplementary Table S1 (Supporting Information), but not shown in Fig. 3) would eliminate 25 data points (4.7%) and result in a remaining average *δ* ^13^C value of −32.34 ± 0.03‰ (1*σ* = 0.68‰, *n* = 509). The latter approach eliminates 30 data points (5.6%), with the remaining 504 data points resulting in an average *δ* ^13^C value of −32.36 ± 0.03‰ (1*σ* = 0.65‰). For the IQR approach, the mean and median are shifted closer to each other, the standard deviation is smaller and the distribution is closer to normal than with the 2*σ* approach and it will therefore be further used in the discussion along with the full dataset of PE analyses by LA/nC/GC/IRMS (Supplementary Table S1; Fig. 3(b)). In either case, however, the value is further away from its certified value.

The histograms of the individual PE analyses suggest two peaks, one on each side of the mean (Figs. 3(a) and 3(b)). One of the maxima coincides with the accepted *δ* ^13^C value of the PE standard, whereas the other is somewhat lower, at around −32.7‰. The observed distribution did not change over the several months involved in this study, but the isotopic values and corresponding yields suggest that this is an actual feature of the data. A difference in distribution is found between analyses based on peak areas smaller and larger than 4 Vs (Figs. 3(c) and 3(d), respectively). Those smaller than 4 Vs show their highest abundance around −32.5‰, but are skewed towards higher values. The mean for this subset is −32.14‰. Interestingly, peak areas larger than 4 Vs are most abundant around the accepted *δ* ^13^C value for PE with a mean of −32.46‰, but are skewed towards lower values. Thus, the mean of each subset coincides with the highest abundance of the other subset. Although the means of the two subsets are different, their deviation is well within the standard deviation of each subset.

**Figure 4:**
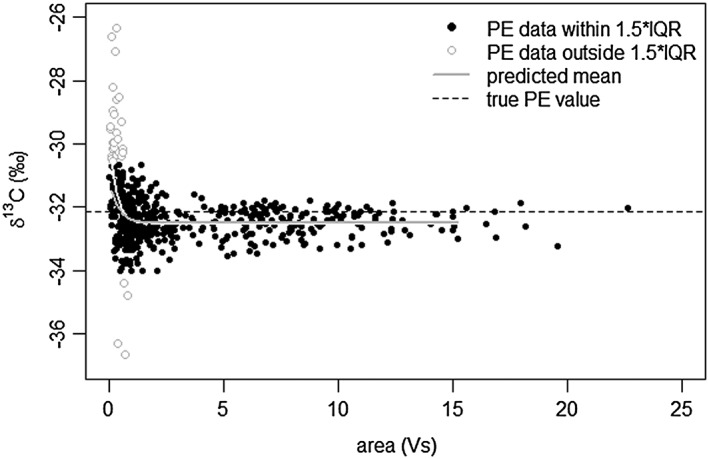
*δ* ^13^ C values versus sample size of all PE measured by LA/nC/GC/IRMS. Sample size reflected by cumulative peak area of *m/z* 44, 45 and 46 expressed in Vs. Predicted mean is white for full PE dataset, grey for IQR dataset. Dashed line is accepted PE value.

In Fig. 4, the *δ* ^13^C values of all PE data measured by LA/nC/GC/IRMS are compared with the peak area. Samples excluded from the IQR dataset all generate peaks under 0.85 Vs and are typically much more enriched in ^13^C. Therefore, these analyses do not represent random outliers.

**Figure 5:**
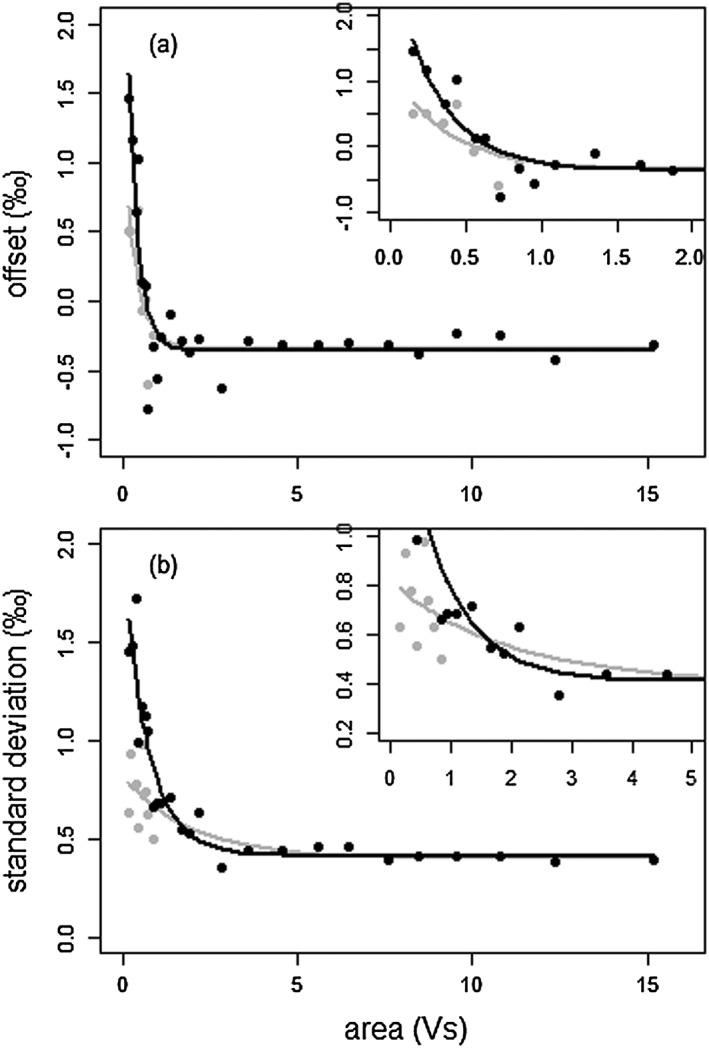
Offset from true PE *δ* ^13^ C value (a) and standard deviation versus peak area (b). Points are calculated values for each of the 25 subsets (see text and Supplementary Table S1, Supporting Information ), lines are the fitted curves. Black is based on the full dataset, whereas grey is for the IQR dataset. Insets are enlargements for a smaller peak area interval.

In order to predict the offset and standard deviation as a function of yield, the full PE dataset and the IQR dataset were each divided into 25 subsets based on peak area (Supplementary Table S2, Supporting Information). We selected the peak area intervals to each consist of roughly 20 data points. Since the data are skewed towards smaller standards, the intervals with small peak areas are narrower and therefore allow for more detailed analysis around the analytical threshold of 0.85 Vs. For each interval, the mean, the offset from the accepted PE value and the standard deviation were calculated and a curve was fitted through these 25 data points (Fig. 5; Supplementary Table S3, Supporting Information). The figure clearly shows, as expected, that the standard deviation and the offset increase at lower yields. The lowest yields show an average offset of almost 1.5‰ towards higher values, with an equally large standard deviation. With increasing yields, the offset decreases and eventually stabilizes at −0.36‰, close to the offset calculated for the entire data set. The fitted curve is more or less stabilized for samples yielding at least 2 Vs. The standard deviation (2*σ*) also decreases with increasing sample size and stabilizes at 0.41‰ for samples generating a peak area of at least 4 Vs. This makes PE analyses yielding a peak area of at least 4 Vs suitable as a standard for correcting values measured on samples in the same setup (i.e. standard bracketing). By experience, a yield of 4 Vs is obtained in our setup when ablating PE for 3 s at 25 kV with an 80 μm crater size.

**Figure 6:**
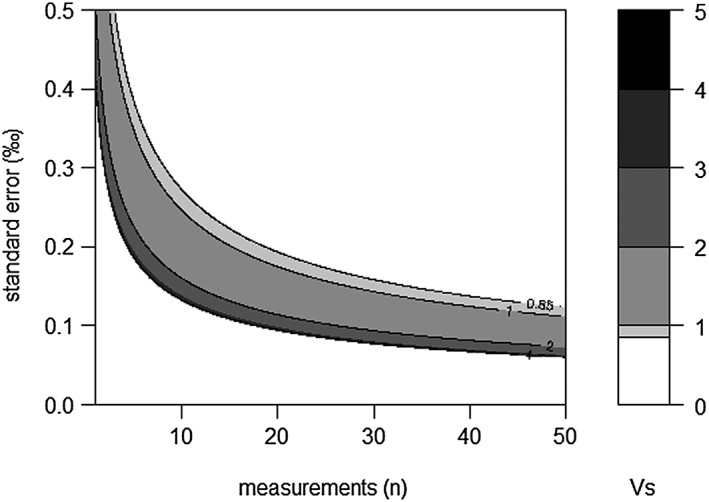
Cumulative peak area of *m/z* 44, 45 and 46 as a function of both standard error and number of measurements for PE. Lines for peak areas of 0.85, 1, 2 and 4 Vs are indicated.

Figure 6 shows the relationship between the peak area, the number of replicates and the standard error, as predicted using the standard deviations of the 25 subsets as a function of the number of replicates within the subset. The standard error decreases both with increasing peak area and with an increasing number of replicates. For example, at least five measurements are required for samples generating peak areas of 4 Vs to achieve a standard error better than 0.2‰, whereas 18 measurements are required for a standard error of less than 0.1‰.

The new setup reaches best precision and accuracy (0.41‰ and 0.36‰, respectively) for single spot analyses yielding more than 4 Vs, which translated to ca 42 ng (3.5 nmol) of carbon. This precision is comparable with those observed by Eek *et al.* 33 and Moran *et al.*,^42^ although the former analyzed soluble organic volatiles rather than solid organic matter (Supplementary Table S4, Supporting Information). Compared with the certified *δ* ^13^C value of the PE standard, analyses of this standard using EA/IRMS show an average offset of 0.43‰ towards lower *δ* ^13^C values. This offset is only slightly larger than that observed for the LA/nC/GC/IRMS PE data (0.41‰) and thus suggests an offset due to the use of different in‐house and international standards and their inter‐calibration. Such an offset is easily overcome by using standard bracketing of pollen analyses using the PE standard.

Based on the fitted curve (Fig. 5(b)), the precision still remains better than 1‰ for samples down to 24 ng C (2*σ*; 2.0 nmol; 2.06 Vs) and for samples down to the analytical threshold of 13 ng C (1.1 nmol; 0.85 Vs) the precision is 1.74‰ (2*σ*). This allows for the analysis of larger single pollen grains. When discussing pollen data, the standard deviation of comparable PE data (i.e. PE data within the same peak area range) should be considered as the predicted internal analytical error. This is, however, assuming homogeneity within the PE standard at this scale. Some heterogeneity in the PE standard would imply a smaller internal error than calculated here and this should therefore be considered as the minimum precision and accuracy for these analyses.

#### **Eucalyptus globulus**

**Figure 7:**
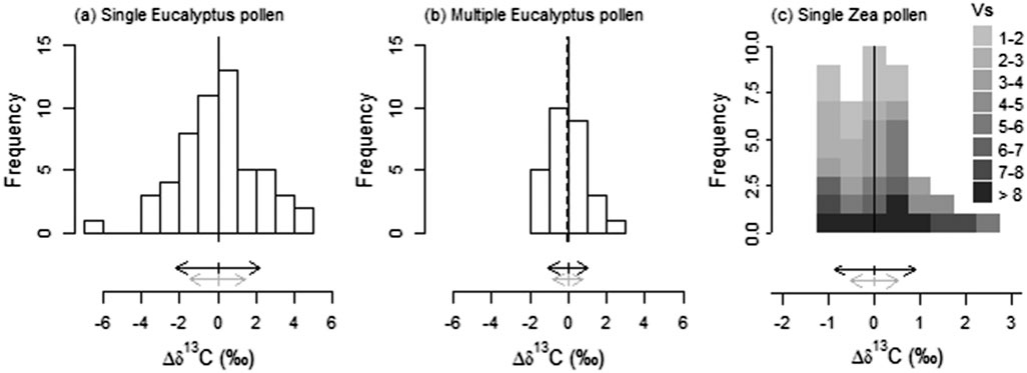
Histograms of Δ *δ* ^13^ C values of Eucalyptus globulus and *Zea mays* pollen. Single *E. globulus* (a), multiple *E. globulus* (b) and single *Z. mays* (c) pollen measurements. Histogram for *Z. mays* is shown in different shades for various area intervals. Vertical line is the mean, dashed line is the median. Black horizontal arrows indicate standard deviation of the mean for pollen. Grey arrows indicate standard deviation of PE samples of 0.199 to 0.742 Vs (single *E. globulus* pollen), 0.505 to 3.160 Vs (multiple *E. globulus* pollen) and 1.118 to 11.498 Vs (single *Z. mays* pollen).

Ablation of a single specimen (a single pollen grain) of the relatively small (22 ± 2 μm diameter) Eucalyptus globulus pollen yielded a signal of between 0.20 and 0.74 Vs, which corresponds to 7–12 ng of carbon based on the calibration in Fig. 2. Although the above PE analyses indicate that such yields are insufficient for robust *δ* ^13^C measurements, we can still compare the results from the assemblage. The mean *δ* ^13^C value is −27.70 ± 0.29‰ (1*σ* = 2.17‰, *n* = 55) and the distribution is close to normal (Shapiro–Wilk *p* = 0.6442, Fig. 7(a)). Despite the large standard deviation, the mean is very close to the −27.85 ± 0.04‰ (1*σ* = 0.07‰, *n* = 4) measured using EA/IRMS using of the order of 2000 specimens per analysis. The mean *δ* ^13^C value of a pollen grain of E. globulus is comparable with that known for bulk plant tissue, leaf tissue, whole wood, twigs, branches, tree rings and tree ring cellulose of the same species, together ranging from −33.3 to −26.1‰.^11^, ^15^, ^18^, ^54^

From the full PE dataset, 113 samples have a peak area within the same range as the single specimen E. globulus analyses, showing a standard deviation of 1.47‰. Based on the fitted curve (Fig. 5(b)), the average peak area of 0.41 Vs corresponds to an expected standard deviation of 1.25‰. The observed standard deviation resulting from single specimen LA/nC/GC/IRMS measurements is 0.70 and 0.92‰ larger than the analytical error predicted by the PE data from the selected peak area interval (Supplementary Table S5, Supporting Information) and the fitted curve (Fig. 5(b)), respectively. This observed larger inter‐specimen variability suggests an additional effect from natural variation between individual pollen grains. However, the calculated inter‐specimen variability should be considered with care because of the relatively large differences in this part of the peak area calibration. Remarkably, the analyses of Nelson^53^ required simultaneous measurement of ten pollen grains of the genera *Ambrosia* and *Artemisia*, which are on average hardly smaller than those of E. globulus, to obtain *m/z* 44 peak areas of 2.5 Vs, corresponding to approximately only 21 ng C*.* This suggests either that not all those ten grains were fully combusted or that a relatively smaller fraction of the obtained CO_2_ reached the IRMS instrument after SWiM than with LA/nC/GC/IRMS.

Because of the limited amounts of CO_2_ produced by ablating single E. globulus pollen, we also analyzed two to five pollen grains simultaneously using LA/nC/GC/IRMS. For these multispecimen pollen analyses, the peak areas of 28 measurements ranged from 0.51 to 3.16 Vs. The mean *δ* ^13^C value of −27.53 ± 0.20‰ (1*σ* = 1.04‰) is 0.17‰ higher than the mean based on measuring individual pollen grains and 0.32‰ higher than the *δ* ^13^C values obtained through EA/IRMS. The Shapiro–Wilk *p*‐value for the multispecimen ablation values distribution is 0.7606, suggesting a normal distribution of the data. The standard deviation for multiple pollen grains is smaller than that of single pollen grain analysis. This implies that inter‐specimen variation is caused to some extent by natural variability, because measuring a weighted average for two to five pollen grains dampens variability between single grains.

From the fitted curve of PE analyses (Fig. 5(b)), the average peak area of 1.54 Vs of the multiple E. globulus pollen would imply an analytical error of 0.59‰ (1*σ*). However, since most pollen analyses actually generated a smaller peak area than the average, this represents an underestimate. From the full PE dataset, 275 samples have a peak area of between 0.51 and 3.16 Vs. Most of these PE data (82%) and most pollen data (82%) are under 2 Vs, thereby being more comparable. The selected PE data have a standard deviation of 0.79‰, which is smaller than in the multiple E. globulus pollen data (Fig. 7(b)). It seems as if the 0.25‰ larger standard deviation of the pollen *δ* ^13^C values than of the PE data may reflect some natural variability between individual pollen grains. Compared with the 0.7‰ variability based on single specimen analyses one would expect a factor of 1/√(n) difference in observed natural variability for multispecimen analyses. These analyses, being based on measuring 2–5 specimens, would then, very roughly, imply a 0.25/√(3.5) = 0.47‰ standard deviation between single pollen grains. This suggests that in addition to single pollen analyses multiple pollen analyses can also, to some extent, be used to assess the natural variability of *δ* ^13^C values between individual pollen grains.

#### **Zea mays**

A number of 43 individual Zea mays pollens (84 ± 8 μm diameter) were analyzed using LA/nC/GC/IRMS. Because of their relatively large size, multiple analyses were performed on twelve individual pollen grains. The sample peaks were between 1.12 and 11.50 Vs in area, reflecting 15 to 110 ng C per ablation, using the calibration based on PE ablations (Fig. 2). The mean *δ* ^13^C value of the population is −11.38 ± 0.14‰ (1*σ* = 0.89‰) and normality is suggested based on a Shapiro–Wilk *p*‐value of 0.2821.

The observed average is 1.65‰ more depleted in ^13^C than the bulk analyses based on measuring on average 40 specimens using EA/IRMS (*δ* ^13^C value of −9.73 ± 0.09‰, 1*σ* = 0.21‰, *n* = 5). This is surprising, as no offset between EA/IRMS and LA/nC/GC/IRMS results was observed for E. globulus, and PE showed an offset in the same direction of only −0.43‰ compared with the bulk analysis. Previous studies in which pollen grains were analyzed for their *δ* ^13^C value consistently show values in line with their C_4_ carbon fixation, albeit that most studies (using EA/IRMS analyses of untreated bulk Z. mays pollen) indicated somewhat lower *δ* ^13^C values than observed here. The *δ* ^13^C values differ considerably between studies: −12.1‰ (*n* = 1),^9^ –10.45‰ (1*σ* = 0.03‰, *n* = 5)^55^ and −10.01‰ (*n* = 1).^10^ Since the different pollen samples were obtained from different regions and at different times, the observed differences in *δ* ^13^C values reflect a large array of possible influences such as temperature, atmospheric CO_2_ concentrations and *δ* ^13^C values, and light intensity.^10^, ^12^, ^14^, ^16^ However, such effects obviously cannot explain the observed offset between bulk and nano combustion devices. Data corrections for organic stable carbon isotope analysis typically include standards with contrasting isotopic composition covering a large range, such as the international standards USGS‐24 (−16.05‰) in addition to the PE standard (IAEA‐CH‐7, −32.15‰), whereas for the LA/nC/GC/IRMS analyses only the PE standard is currently available. While E. globulus has isotopic values only approximately 4 to 6‰ more enriched in ^13^C compared with the reference gas and PE, Z. mays is much more enriched – by approximately 22–26‰. A small error in calibration and/or correction factor could hence be much more amplified over the large isotopic range between standard and sample. A ^13^C–enriched standard would therefore be preferred to correct individual Z. mays values. Thus far, however, no suitable material with a higher *δ* ^13^C value has proven stable and homogenous enough to be used as a standard on the nanogram scale.

Although Z. mays carbon isotopic ratios based on LA/nC/GC/IRMS are systematically offset from the EA/IRMS bulk value, the data still allow the study of population variability (Fig. 7(c)). Although the histogram suggests a bimodal distribution, Hartigan's dip test does not reject the hypothesis of the data being unimodally distributed (*p* = 0.2232). In addition, no systematic offset is observed between samples with high or low yields (Fig. 7(c)). The standard deviation of all Z. mays (0.89‰) is larger than the fitted curve for the standard deviation of PE at 4.67 Vs, which is the average peak area for the Z. mays analyses (0.42‰, Fig. 5(b)). The pollen data are, however, probably best compared with the PE data itself rather than with the fitted curve as both datasets contain a considerable number of samples that are smaller than the average peak area applied in the fitted curve. Direct comparison with peak areas within the same range implies a maximum standard deviation of 0.52‰ for the PE standard. To double check whether a size effect could still affect the observed offset in standard deviations between PE and Z. mays pollen, we separated the data in four subsets based on their peak area (Supplementary Table S5, Supporting Information). As expected, the standard deviation of PE is highest for the smallest yields and becomes more or less stable around 0.4‰ for samples over 3 Vs. The standard deviation of Z. mays pollen shows an opposite effect at the lowest yield, with a 0.52‰ standard deviation for the smallest samples, and a constant 1.0‰ standard deviation for samples over 3 Vs. Although the low standard deviation at the lowest yield is unexpected, overall the difference from the PE standard suggests that approximately 0.4‰ of the observed variation between Z. mays pollen is due to the analytical uncertainty and the remaining approximately 0.6‰ is due to natural inter‐pollen variability. In addition, when comparing the standard error of the Z. mays analyses with the calculated uncertainty, it is clear that the values do not drop below 0.2‰ as would be expected when analyzing ten standards larger than 5 Vs (Supplementary Table S6, Supporting Information; Fig. 6).

**Figure 8:**
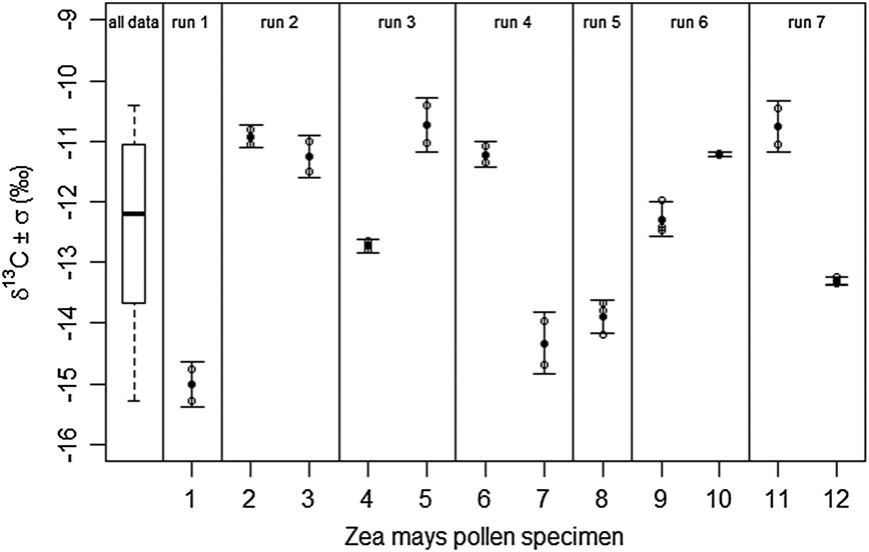
Replicate measurements of *δ* ^13^ C values on twelve *Zea mays* pollen grains. Boxplot shows distribution of all measurements on twelve pollens. Open circles reflect two or three measurements per single pollen, closed circles indicate the mean *δ* ^13^ C for each pollen. Error bars indicate standard deviations.

Because of their relatively large size, twelve Z. mays pollen grains were each measured individually two or three times (Fig. 8). This potentially allows the isotopic variability within a single pollen grain to be investigated. The peak areas range from 0.62 to 9.20 Vs (11–89 ng C). The average *δ* ^13^C value for all analyses is −12.36 ± 0.29‰ and the standard deviation is 1.48‰ (1*σ*). The average value seems somewhat offset from the other analyses, and the overall standard deviation seems somewhat higher. However, although the number of analyses is substantial (*n* = 26), the number of pollen grains analyzed here was relatively limited. Therefore, we here focus on the variability within the pollen grains. The standard deviation of the analyses within individual pollen grains is 0.27 ± 0.04‰ (Fig. 8), which is similar to the 0.21‰ standard deviation of the bulk EA/IRMS measurements, and even somewhat smaller than the 0.41‰ standard deviation of PE ablations with high yield. This shows that there is no difference in *δ* ^13^C values within pollen grains at this scale and that the measurements of pollen are at least as precise as for the PE standard. This potentially even indicates that a single Z. mays pollen grain is more homogeneous than the PE foil at the scale considered here. The precise replication of the pollen *δ* ^13^C value also supports the observation that variation between pollen grains is not solely due to analytical errors. The intra‐pollen differences plotted together in a single frame (Fig. 8) show that inter‐pollen offsets are not due to drift and that the inter‐pollen variability is much larger than the intra‐pollen variability.

### Outlook and implications

At the pollen grain level, the *δ* ^13^C values clearly allow us to distinguish between C_3_ and C_4_ plants.^9^, ^10^ When combining all single pollen grains, multiple pollen grains and replicate analyses for both E. globulus on one hand and for Z. mays on the other, we find a 7.54‰ gap between the highest E. globulus *δ* ^13^C value and the lowest Z. mays *δ* ^13^C value, thereby clearly separating the two data populations. The mean *δ* ^13^C value of all Z. mays data is 16‰ higher than the mean *δ* ^13^C value of the E. globulus data. Although this result was 18.12‰ using traditional EA/IRMS and an additional standard more enriched in ^13^C would be required to thoroughly calibrate the Z. mays data, this difference is similar to the 19.3‰ offset found between leaf *δ* ^13^C values of Z. mays and E. globulus,^18^ which suggests no large additional differences in isotopic fractionation between plant and pollen for these species. The method shown here can, however, analyze much smaller fragments than plant leaves and would potentially also allow us to distinguish subtler differences between the isotopic signatures of different pollens and hence plants that result from the *δ* ^13^C dependence on environmental variables.

Isotopic variability between individual pollen samples within a species has been suggested previously by bulk pollen analysis of various species,^10^, ^56^, ^57^ but so far the only analyses on single pollen grains focus solely on distinguishing between C_3_ and C_4_ plants.^56^, ^58^ Bulk samples, each consisting of pollen from one to ten plants of a single species, with the samples obtained from different locations and sampled at different times, showed a range in *δ* ^13^C values within a species of up to 6‰.^10^ Since both the E. globulus pollen and the Z. mays pollen samples potentially consist of pollen from multiple plants, the natural variability in the pollen is not known either spatially or temporally. However, the observed variability shows that the range in isotopic values of E. globulus is very limited and is only slightly larger in Z. mays, which suggests that the pollens are from a homogeneous and/or limited population. However, the *δ* ^13^C values of single pollen grains can be used to relate them to environmental variables, thus providing a new and unique tool for forensics, geology and ecology. Furthermore, although we did not observe isotopic variability within pollen specimens, the approach as such is very much suitable to be applied to other species, which might show variable *δ* ^13^C values within a single specimen.

In addition to untreated modern pollen grains, we successfully analyzed other substrates such as sub‐fossil and fossil dinoflagellate cysts, lacustrine ephippia and fossil phytoclasts. The preliminary results (not shown) suggest that the setup presented here is suitable for all kinds of small organic particles, such as spores, seeds, dinoflagellates, dinoflagellate cysts, acritarchs, organic foraminiferal linings, tissues, larger animal and plant cells, chitinozoans, scolecodonts; anything organic that can properly be disintegrated by the ArF laser. These analyses allow for comparison between and within these particles, depending on their size.

For geological applications it remains necessary to also consider taphonomical effects, such as those related to partial oxidation and diagenesis,^59^ as well as chemical treatments sometimes used for the isolation of organic fossils. For pollen grains, for example, it was found that the *δ* ^13^C value changes appreciably when only the extracted sporopollenin or fossil remnants of pollen (diagenetically altered sporopollenin) are analyzed instead of untreated grains.^10^, ^60^ Because larger pollen grains still contain sufficient carbon in the shape of sporopollenin to be successfully analyzed for their isotopic composition, investigation of such taphonomical effects would be within reach of the here proposed method. The new setup therefore thus sets the stage for investigating numerous questions from modern ecology, to preservation and taxonomy, and, considering recent biogeological experiments,^61^ potentially even carbon cycling.

## Conclusions

We present a novel LA/nC/GC/IRMS setup capable of measuring *δ* ^13^C values with a precision of 1‰ (2*σ*) for samples down to 24 ng C (2.06 Vs) based on repeated analyses of an international PE standard. Analyses with a larger ablation yield (from 4 Vs, 42 ng C) show the optimal standard deviation achievable with this setup of 0.41‰, which approaches other bulk analytical techniques. These standard deviations indicate the maximum analytical errors as the PE foil is possibly slightly heterogeneous at the small scale here considered.

Application of the new approach shows that a single untreated modern pollen grain could successfully be analyzed. The average *δ* ^13^C values of E. globulus and Z. mays pollen differ by ~16‰, thereby clearly distinguishing between the isotopic signatures of C_3_ and C_4_ plants. However, an additional standard with a higher *δ* ^13^C value is required to allow for more accurate calibrations, especially for samples with *δ* ^13^C values deviating further from that of PE, but this is currently unavailable. Pollen grains of E. globulus yielded only 7–12 ng C in the IRMS instrument, which is insufficient for accurate analysis. As a result, there is no solid proof that the 0.70‰ variability recorded in excess of the analytical error at this range of yields truly reflects inter‐specimen variability. Average *δ* ^13^C values measured using LA/nC/GC/IRMS agree well with the EA/IRMS results.

Analyses of different parts of the same Z. mays pollen grain show identical isotopic compositions within the analytical error. Between single pollen grains more variability in *δ* ^13^C values is observed, which appears to account for 0.4–0.6‰ of natural variability on top of the analytical error. These results suggest that the presented LA/nC/GC/IRMS setup could be used to further investigate within‐population variability for pollen grains as well as other small‐scale carbon‐containing particles in geology, biology and other research fields.

## Supporting information


**Fig. S1.** LA/nC/GC/IRMS system setup*.* Lines represent fused silica capillary (length and inner diameter are indicated) connecting helium supply from a GCIII interface to the ablation chamber, through the oxidation oven and a GC column and via the nafion and open split of the GCIII interface to the IRMS instrument. Thick black line in the center of the ablation chamber represents the nickel disc on which the sample and standard are positioned.
**Fig. S2.** Photo of the ablation chamber. Top view (a) and front view (b). Chamber dimensions are described in the main article.
**Fig. S3.** Example of typical run output showing peak intensity (mV) versus retention time (s). First and last three peaks correspond to reference gas injections. Two PE standards are followed by two Z. mays samples and another PE standard. Signal intensity is given in mVs. Peaks are integrated (not shown) according to default settings, namely between an ascending slope of 0.2 mV/s and a decreasing slope of 0.4 mV/s.
**Table S1.** Datasets of PE measured by LA/nC/GC/IRMS. Mean, median, standard deviation, number of measurements, standard error, minimum and maximum *δ* ^13^C values and *p*‐value resulting for Shapiro–Wilk normality test (α <0.05) of all PE data, PE data selected by eliminating data outside the 2*σ* and PE data selected by eliminating data outside 1.5 times the interquartile range. Histograms of all PE data and PE data selected by eliminating data outside 1.5 times the interquartile range are shown in Fig. 3a,b in the main article. PE data within 2*σ* are not further discussed or shown.
**Table S2.** PE characteristics per peak area based subset. Mean *δ* ^13^C value, offset from accepted PE value, standard deviation and number of measurements for each subset are given.
**Table S3.** Variables based on fitted curve (Fig. 5) at given peak size, model slope and standard deviation. Theoretical offset in *δ* ^13^C values (‰) and standard deviation of *δ* ^13^C values (*σ* in ‰) predicted for minimum and maximum sample size (Vs = 0, ∞) , and required peak size (Vs) determined for slope of the model being smaller than 0.01 ‰/Vs and for a given *σ.*
**Table S4.** Comparison of obtained precision of *δ* ^13^C analyses for the various methods as discussed in the main article. Analyzed amount of carbon expressed in both ng C and nmol C. Precision is given for 2*σ*. The main substrate for which each method was primarily designed is also listed.
**Table S5.** Standard deviation of *δ* ^13^C values, number of measurements and standard error of *δ* ^13^C values for Eucalyptus globulus and PE. Single pollen data and corresponding PE subset (a), and multiple pollen data with corresponding PE subset (b) and multiple pollen with corresponding PE subset.
**Table S6.** Standard deviation of *δ* ^13^C values, number of measurements and standard error of *δ* ^13^C values for Zea mays and PE. All single pollen data with corresponding PE subset (a) and single pollen and PE data separated into four peak area ranges (b).

Supporting info item
