# Supplementary material for: Stable carbon isotope analyses of nanogram quantities of particulate organic carbon (pollen) with laser ablation nano combustion gas chromatography/isotope ratio mass spectrometry
Source: Rapid Commun Mass Spectrom. 2016 Nov 28;31(1):47–58. doi: 10.1002/rcm.7769 (PMC5132107; doi:10.1002/rcm.7769)
Supplement: Supplementary file 1 — Supporting info item [file RCM-31-47-s001.docx]

**SUPPORTING INFORMATION**

**Stable carbon isotope analyses of nanogram quantities of particulate organic carbon (pollen) with laser ablation nano combustion gas chromatography/isotope ratio mass spectrometry**

**Linda van Roij^1^*, Appy Sluijs^1^, Jelmer J. Laks^1^ and Gert-Jan Reichart^1,2^**

^1^Department of Earth Sciences, Faculty of Geosciences, Utrecht University, Heidelberglaan 2, 3584 CS Utrecht, The Netherlands

^2^Royal Netherlands Institute for Sea Research (NIOZ), Landsdiep 4, 1797 SZ 't Horntje (Texel), The Netherlands

**Correspondence to*: L. van Roij, Department of Earth Sciences, Faculty of Geosciences, Utrecht University, Heidelberglaan 2, 3584 CS Utrecht, The Netherlands.

E-mail: L.vanRoij@uu.nl; lindavanroij@gmail.com

This is an open access article under the terms of the Creative Commons Attribution-NonCommercial-NoDerivs License, which permits use and distribution in any medium, provided the original work is properly cited, the use is non-commercial and no modifications or adaptations are made.


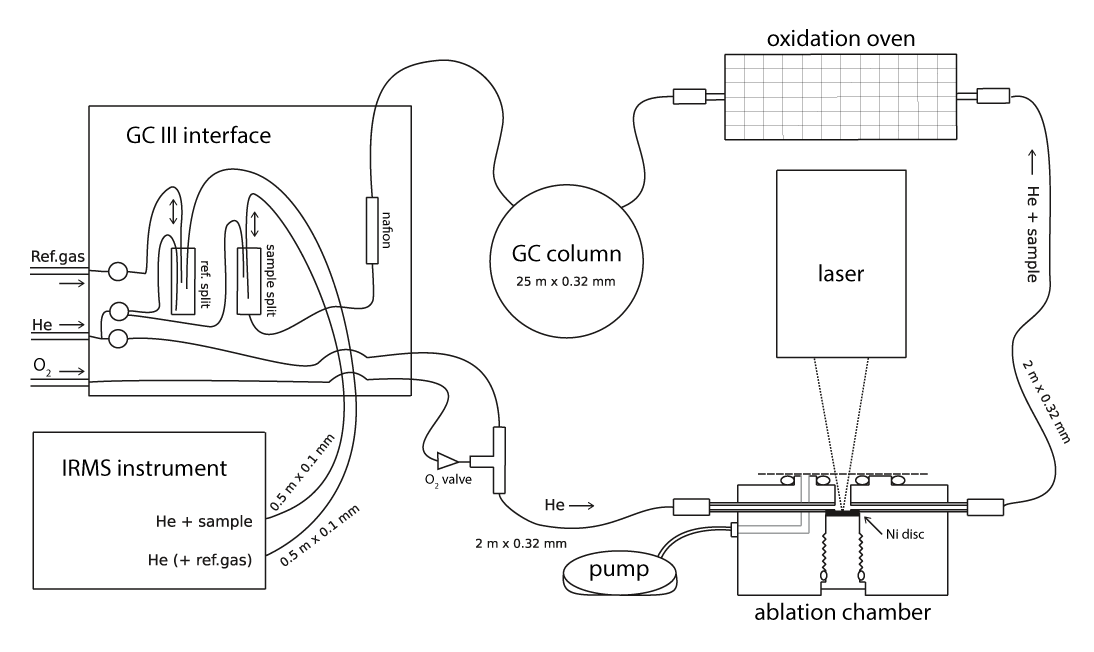


**Figure S1.** LA/nC/GC/IRMS system setup*.* Lines represent fused silica capillary (length and inner diameter are indicated) connecting helium supply from a GCIII interface to the ablation chamber, through the oxidation oven and a GC column and via the nafion and open split of the GCIII interface to the IRMS instrument. Thick black line in the center of the ablation chamber represents the nickel disc on which the sample and standard are positioned.


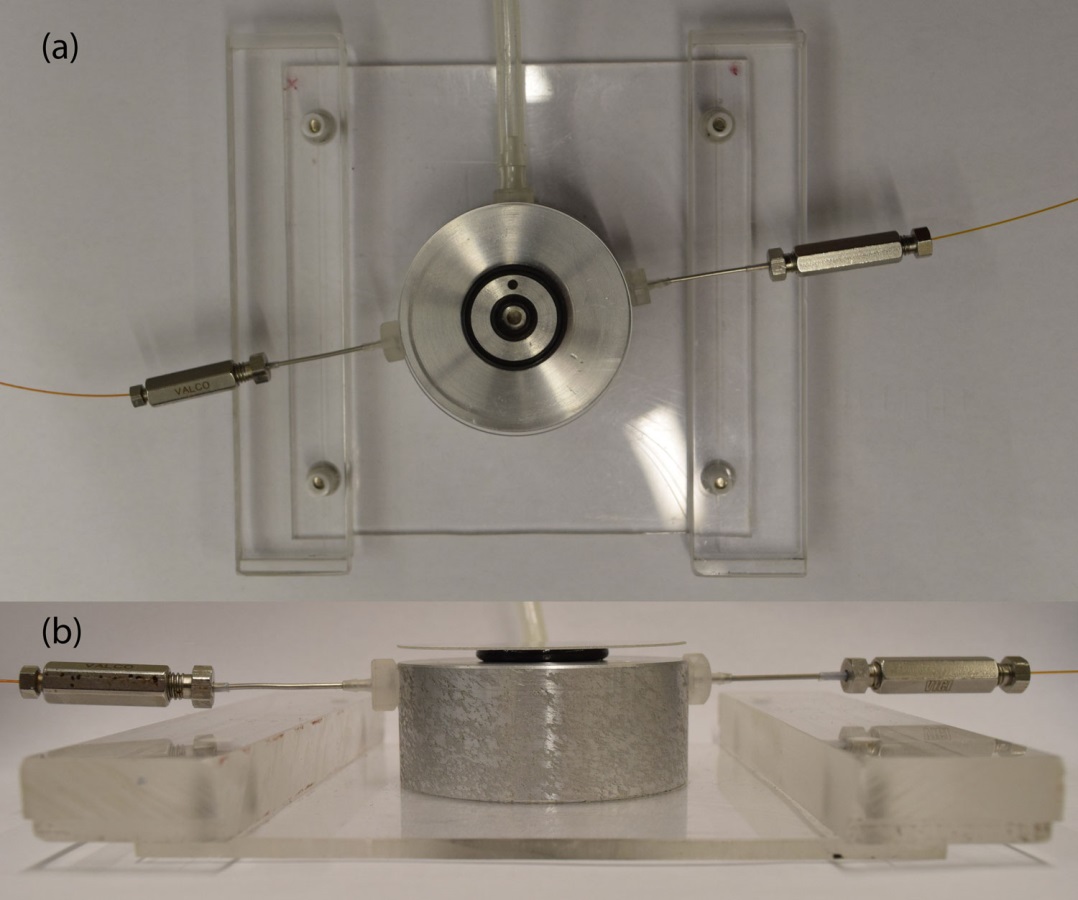


**Figure S2.** Photo of the ablation chamber. Top view (a) and front view (b). Chamber dimensions are described in the main article.


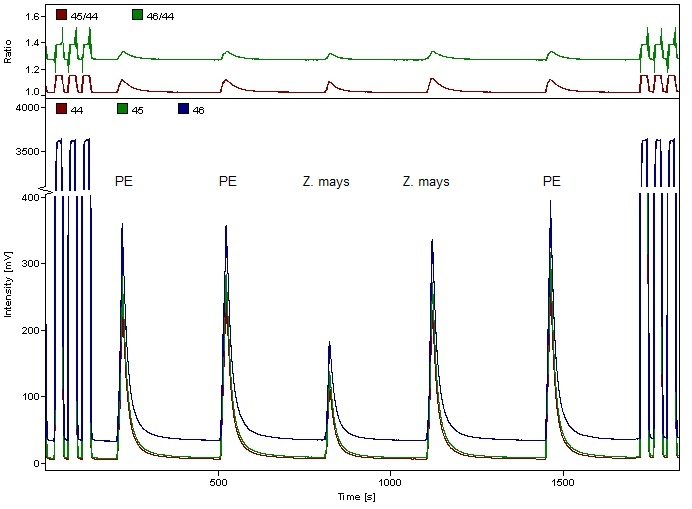


**Figure S3.** Example of typical run output showing peak intensity (mV) versus retention time (s). First and last three peaks correspond to reference gas injections. Two PE standards are followed by two *Z. mays* samples and another PE standard. Signal intensity is given in mVs. Peaks are integrated (not shown) according to default settings, namely between an ascending slope of 0.2 mV/s and a decreasing slope of 0.4 mV/s.

**Table S1.** Datasets of PE measured by LA/nC/GC/IRMS. Mean, median, standard deviation, number of measurements, standard error, minimum and maximum *δ*^13^C values and p-value resulting for Shapiro-Wilk normality test (α <0.05) of all PE data, PE data selected by eliminating data outside the 2*σ* and PE data selected by eliminating data outside 1.5 times the interquartile range. Histograms of all PE data and PE data selected by eliminating data outside 1.5 times the interquartile range are shown in Fig. 3a,b in the main article. PE data within 2*σ* are not further discussed or shown

| **PE** | **All data** | **Within 2*σ*** | **Within 1.5*IQR** | **Certified** |
| --- | --- | --- | --- | --- |
| mean *δ*^13^C | -32.24 | -32.34 | -32.36 | -32.151 |
| median *δ*^13^C | -32.33 | -32.36 | -32.37 |  |
| *σ* | 0.98 | 0.68 | 0.65 | 0.05 |
| n | 534 | 509 | 504 |  |
| error | 0.04 | 0.03 | 0.03 |  |
| min *δ*^13^C | -36.66 | -34.29 | -34.10 |  |
| max *δ*^13^C | -26.34 | -30.37 | -30.55 |  |
| Shapiro-Wilk (p) | 0.0000 | 0.0013 | 0.0168 |  |

**Table S2.** PE characteristics per peak area based subset. Mean *δ*^13^C value, offset from accepted PE value, standard deviation and number of measurements for each subset are given

|  | All PE data | | | | PE data within 1.5IQR | | | |
| --- | --- | --- | --- | --- | --- | --- | --- | --- |
| **area (Vs)** | **mean *δ*^13^C** | **offset** | ***σ*** | **n** | **mean *δ*^13^C** | **offset** | ***σ*** | **n** |
| 0 - 0.2 | -30.69 | 1.46 | 1.45 | 23 | -31.65 | 0.50 | 0.63 | 13 |
| 0.2 - 0.3 | -30.99 | 1.16 | 1.48 | 18 | -31.64 | 0.51 | 0.93 | 13 |
| 0.3 - 0.4 | -31.50 | 0.65 | 1.72 | 27 | -31.79 | 0.36 | 0.78 | 22 |
| 0.4 - 0.5 | -31.13 | 1.02 | 0.98 | 16 | -31.49 | 0.66 | 0.55 | 13 |
| 0.5 - 0.6 | -32.01 | 0.14 | 1.17 | 23 | -32.22 | -0.07 | 0.98 | 21 |
| 0.6 - 0.7 | -32.03 | 0.12 | 1.12 | 16 | -32.14 | 0.02 | 0.73 | 13 |
| 0.7 - 0.8 | -32.93 | -0.78 | 1.05 | 21 | -32.75 | -0.60 | 0.63 | 20 |
| 0.8 - 0.9 | -32.48 | -0.33 | 0.66 | 29 | -32.40 | -0.25 | 0.50 | 28 |
| 0.9 - 1 | -32.71 | -0.56 | 0.68 | 24 | -32.71 | -0.56 | 0.68 | 24 |
| 1 - 1.25 | -32.42 | -0.26 | 0.68 | 41 | -32.42 | -0.26 | 0.68 | 41 |
| 1.25 - 1.5 | -32.24 | -0.09 | 0.71 | 35 | -32.24 | -0.09 | 0.71 | 35 |
| 1.5 - 1.75 | -32.44 | -0.29 | 0.55 | 20 | -32.44 | -0.29 | 0.55 | 20 |
| 1.75 - 2.0 | -32.52 | -0.36 | 0.52 | 22 | -32.52 | -0.36 | 0.52 | 22 |
| 2.0 - 2.5 | -32.43 | -0.28 | 0.63 | 25 | -32.43 | -0.28 | 0.63 | 25 |
| 2.5 - 3 | -32.78 | -0.63 | 0.35 | 17 | -32.78 | -0.63 | 0.35 | 17 |
| 3 - 4 | -32.43 | -0.28 | 0.44 | 10 | -32.43 | -0.28 | 0.44 | 10 |
| 4 - 5 | -32.46 | -0.31 | 0.44 | 15 | -32.46 | -0.31 | 0.44 | 15 |
| 5 - 6 | -32.47 | -0.32 | 0.46 | 24 | -32.47 | -0.32 | 0.46 | 24 |
| 6 - 7 | -32.45 | -0.30 | 0.46 | 28 | -32.45 | -0.30 | 0.46 | 28 |
| 7 - 8 | -32.46 | -0.31 | 0.39 | 21 | -32.46 | -0.31 | 0.39 | 21 |
| 8 - 9 | -32.54 | -0.39 | 0.41 | 17 | -32.54 | -0.39 | 0.41 | 17 |
| 9 - 10 | -32.38 | -0.23 | 0.41 | 15 | -32.38 | -0.23 | 0.41 | 15 |
| 10 - 12 | -32.40 | -0.25 | 0.41 | 20 | -32.40 | -0.25 | 0.41 | 20 |
| 12 - 14 | -32.58 | -0.43 | 0.38 | 10 | -32.58 | -0.43 | 0.38 | 10 |
| > 14 | -32.46 | -0.31 | 0.39 | 17 | -32.46 | -0.31 | 0.39 | 17 |
| total |  |  |  | 534 |  |  |  | 504 |

**Table S3.** Variables based on fitted curve (Fig. 5) at given peak size, model slope and standard deviation. Theoretical offset in *δ*^13^C values (‰) and standard deviation of *δ*^13^C values (*σ* in ‰) predicted for minimum and maximum sample size (Vs = 0, ∞) , and required peak size (Vs) determined for slope of the model being smaller than 0.01 ‰/Vs and for a given *σ*

|  | All PE data | PE data within 1.5*IQR |
| --- | --- | --- |
| Largest samples (Vs = ∞) | | |
| Offset (‰) | -0.36‰ | -0.35‰ |
| *σ* (‰) | 0.41‰ | 0.40‰ |
| Smallest samples (Vs = 0) | | |
| Offset (‰) | 2.83‰ | 1.16‰ |
| *σ* (‰) | 1.88‰ | 0.82‰ |
| Slope of fitted model < 0.01 ‰/Vs | | |
| Offset | 2.11 Vs | 2.26 Vs |
| *σ* | 3.87 Vs | 5.95 Vs |
| Variable peak sizes at given σ | | |
| *σ* = 1‰ | 0.67 Vs | NA, maximum *σ* of 0.82 Vs |
| *σ* = 0.5‰ | 2.06 Vs | 2.73 Vs |

**Table S4.** Comparison of obtained precision of *δ*^13^C analyses for the various methods as discussed in the main article. Analyzed amount of carbon expressed in both ng C and nmol C. Precision is given for 2*σ*. The main substrate for which each method was primarily designed is also listed

| **Study** | **Method** | **ng C** | **nmol C** | **Precision** (2*σ*) | **Substrate** |
| --- | --- | --- | --- | --- | --- |
| Werner *et al.* (1999) [24] | EA/IRMS | 25000 | 2083 | <0.2‰ | Organic and inorganic solids and liquids |
|  |  | 2000 | 167 | 1.6‰ |  |
|  |  | 1000 | 83 | 1.8‰ |  |
| Polissar *et al.* (2008) [28] | Nano/EA | 500 | 41 | 1‰ | Organic and inorganic solids and liquids |
| Sessions *et al.* (2005) [34] | SWiM/IRMS | 120 | 10 | 0.4‰ | Soluble nonvolatile organic carbon |
|  |  | 12 | 1 | 2‰ |  |
| Eek *et al.* (2007) [33] | SWiM/IRMS | 50-100* | 4-8 | <0.2‰ | Organic solids, microbial cells |
|  |  | 10* | 0.8 | 1.0‰ |  |
| Moran *et al.* (2011) [42] | LA/IRMS | 65 | 5.4 | 0.2-0.5‰ | Organic solids, spatial resolution |
| This study | LA/IRMS | 42 | 3.5 | 0.41‰ | Organic solids, single pollen |
|  |  | 24 | 2.0 | 1.00‰ |  |
|  |  | 13 | 1.1 | 1.74‰ |  |

* ng C per µL solution

**Table S5.** Standard deviation of *δ*^13^C values, number of measurements and standard error of *δ*^13^C values for *Eucalyptus globulus* and PE. Single pollen data and corresponding PE subset (a), and multiple pollen data with corresponding PE subset (b) and multiple pollen with corresponding PE subset

|  |  | *E. globulus* pollen | | | PE subset | | |
| --- | --- | --- | --- | --- | --- | --- | --- |
| Area (Vs) |  | *σ* (‰) | n | SE | *σ* (‰) | n | SE (‰) |
| 0.199 - 0.742 | (a) | 2.16 | 55 | 0.29 | 1.47 | 113 | 0.14 |
| 0.505 - 3.160 | (b) | 1.04 | 28 | 0.20 | 0.79 | 275 | 0.05 |

**Table S6.** Standard deviation of *δ*^13^C values, number of measurements and standard error of *δ*^13^C values for *Zea mays* and PE. All single pollen data with corresponding PE subset (a) and single pollen and PE data separated into four peak area ranges (b)

|  |  | *Zea mays* pollen | | | PE subset | | |
| --- | --- | --- | --- | --- | --- | --- | --- |
| Area (Vs) |  | *σ* (‰) | n | SE | *σ* (‰) | n | SE (‰) |
| 1.118 - 11.498 | (a) | 0.89 | 43 | 0.14 | 0.52 | 295 | 0.03 |
| 1-3 | (b) | 0.52 | 15 | 0.13 | 0.63 | 160 | 0.05 |
| 3-5 |  | 0.99 | 10 | 0.31 | 0.43 | 25 | 0.09 |
| 5-7 |  | 0.97 | 10 | 0.32 | 0.46 | 52 | 0.06 |
| > 7 |  | 1.03 | 8 | 0.34 | 0.39 | 100 | 0.04 |
